# Notes and News

**Published:** 1897-06-05

**Authors:** 


### NOTES AND NEWS.

(Collected and Communicated.)

Mr. Appleby, of Accrington, is presenting £1,000 to the cottage hospital in commemoration of the present reign.

One of the galleries in the new building of the Brompton Hospital for Consumption is to be named the York Gallery, by permission of the Duke of York.

The Dowager Lady Loder has made a donation of £1,000 to Charing Cross Hospital for the endowment in perpetuity of a bed in memory of the late Sir Robert Loder, M.P.

The Baroness de Hirsch, among her many recent munificent presents to charities, has made a donation of £4,000 to the Prince of Wales's Hospital Fund. The Trustees of the Peabody Fund have also made the handsome donation of £10,000 to the Prince's Fund.

The Salutaris Water Company has offered to present annually to each of the following hospitals fifty quarts, or equivalent quantities of smaller-sized bottles, of Salutaris Water for use in their free wards: St. Thomas's, London, Middlesex, St. George's, Guy's, St. Bartholomew's, St. Mary's, University College, Charing Cross, King's College, Westminster, and the Royal Free.

The Metropolitan Water Companies Bill, which was read a first time on Monday, May 24th, is a temporary measure, to serve until the Royal Commission shall have reported. Its chief practical effect will be to enable the local authorities to initiate complaints when members of their constituencies are aggrieved, instead of this duty being thrown, as at present, on the consumer.

We have been recently consulted in the matter of a scheme to commemorate the Queen's long reign, proposed by one of our readers, which we think so excellent in conception that it is well it should be made more widely known. A lady, desiring to make her offerings to charitable institutions through her grandchildren, proposes to pay certain sums to various institutions in the names of the children, this money to accumulate so that at a certain age it will amount to the sum which will render each child a life governor of the chosen institution. In this way quite a moderate sum can be so distributed as to render material assistance to the charities to be benefited, and be the means of exciting a genuine interest in them on the part of the young people one day to be associated in the work of the institutions.

In connection with the Seamen's Hospital, Greenwich, a scheme is proposed to raise a special fund of £10,000 for the benefit of sick mariners as a national act of commemoration.

It is announced that July 25th will be the date chosen for collections throughout the churches in aid of the Clergy Sustentation Fund. Some confusion has arisen on the subject of the date of Hospital Sunday and the Clergy Sustentation Fund, but matters are now settled.

A great desire is expressed at Perth that a consumption hospital should be established for the town and district. The mortality from consumption in Perth is very considerable, amounting to 10 per cent. of the total deaths in the town, and there is no adequate accommodation for these cases.

The hospitals and charities in general of Naples are likely to undergo a much severer scrutiny than they have been subject to for many years past through the alleged scandals revealed at the Hospital of the Annunziata. There are serious charges now made against the management, who, it is alleged, have allowed the institution to tumble into ruin and become generally disorganised. The head of the institution, it appears, has been living at Rome, and, therefore quite unable to properly attend to the administration. Great excitement has been caused in Naples by the revelations, as the carelessness with which those in authority are charged relates principally to young children, who are received in the hospital.

Dr. T. E. Hayward, Medical Officer of Health, Haydock Urban District Council, has drawn up a very careful annual report. Among the many points of interest raised by the statistical tables which he publishes we will only allude to two. In one table he gives the death-rate from various diseases in every year from 1838 to the present time. From this we find that although diphtheria has been much more prevalent during the last two years than for many years before it has not caused anything like the mortality which it has done on several occasions previously. Last year it produced a death-rate of 0·38 per 1,000 persons living; but in 1863 the rate was 3·81; in 1864, 2·44; and in 1868, 3·36 per 1,000. The average death-rate from diphtheria for England and Wales during the past ten years is given as 0·21 per 1,000. From this we may judge how virulent this disease has been at times in certain districts in times gone by. The next point to which we would allude is the high and increasing puerperal mortality in the Haydock district. The death-rate both from puerperal fever and from accidents of child-birth has been far higher during the last twenty-five years than during the preceding quarter of a century, so much so that the combined mortality from both causes has been more than twice as high between 1871 and 1895 as it was between 1847 and 1870, and far higher than in the rest of England. In the five years from 1891 to 1895 the maternal mortality from these two causes amounted to 11·85 per 1,000 children born alive, the corresponding rate for England and Wales being 5·50. This seems to be a matter worthy of the most careful investigation.

